# When two become one: perceptual completion in pea plants

**DOI:** 10.1080/15592324.2025.2473528

**Published:** 2025-03-13

**Authors:** Silvia Guerra, Bianca Bonato, Laura Ravazzolo, Marco Dadda, Umberto Castiello

**Affiliations:** a Department of General Psychology, University of Padova, Padova, Italy; b Department of Agronomy, Food, Natural Resources, Animals and Environment (DAFNAE), University of Padova, Agripolis, Padova, Italy

**Keywords:** Approach-to-clasp movement, kinematical analysis, perceptual completion, *Pisum sativum* L., plant behavior, plant movement, climbing plants

## Abstract

Pea plants depend on external structures to reach the strongest light source. To do this, they need to perceive a potential support and to flexibly adapt the movement of their motile organs (e.g. tendrils). In natural environments, there are several above- and belowground elements that could impede the complete perception of potential supports. In such instances, plants may be required to perform a sort of perceptual “completion” to establish a unified percept. We tested whether pea plants are capable of performing perceptual completion by investigating their ascent and attachment behavior using three-dimensional (3D) kinematic analysis. Pea plants were tested in the presence of a support divided into two parts positioned at opposite locations. One part was grounded and perceived only by the root system. The remaining portion was elevated from the ground so that it was only accessible by the aerial part. Control conditions were also included. We hypothesized that if pea plants are able to perceptually integrate the two parts of the support, then they would perform a successful clasping movement. Alternatively, if such integration does not occur, plants may exhibit disoriented exploratory behavior that does not lead to clasping the support. The results demonstrated that pea plants are capable of perceptual completion, allowing for the integration of information coming from the root system and the aerial part. We contend that perceptual completion may be achieved through a continuous crosstalk between a plant’s modules determined by a complex signaling network. By integrating these findings with ecological observations, it may be possible to identify specific factors related to support detection and coding in climbing plants.

## Introduction

Plants have evolved a variety of adaptive behaviors to face their sessile lifestyle. Based on growth, plants exhibit numerous movements to explore, navigate and colonize their environment.^1,2^ For instance, climbers (i.e., non-self-supporting plants) adopt circumnutation, a reversible oscillatory growth movement, to explore their surroundings in the search for useful supports.^1–9^

In this connection, recent studies have proved that climbing plants not only are able to sense the presence of potential supports in the environment but also to adapt the morphology and the kinematics of their tendrils (i.e., the filamentary organs climbers use to clasp a support, such as modified leaflets in *Pisum sativum* L.), depending on the chosen support’s physical properties (e.g., thickness.^10–13^

A variety of hypotheses regarding the mechanisms underlying these adaptations have been proposed.^1,3,14–18^ Among these, a plausible candidate is the functional equilibrium subtended by the continuous crosstalk between the plant’s grounded (i.e., the root system) and the aerial (i.e., stem and tendrils) sections via hormonal and/or electrical signaling.^14,19–24^ By means of the scouting of root exudates^15^ and/or mechanical stimulation (i.e., the roots touching the belowground part of the support,^1^ the root system may act as a navigator providing the plant’s aerial section with continuous information regarding the position and the nature of the support. Then, the ascent and clasp behaviors are organized accordingly. Evidence supporting this assertion comes from observations that pea plants are unable to code for the position and properties of a potential support without the sensory inputs the roots provide.^25^

In this perspective, it is assumed that the above- and the belowground sections of the plant should perceive the support to organize an optimal ascent and clasping behavior. However, environmental conditions frequently include several elements, above- and belowground, which may hinder the full perception of the support. In these circumstances, plants may need to perform a sort of perceptual “completion” to reconstruct the unperceivable part of the support and build a unified percept. Perceptual “completion” refers to the process of bridging the gaps imposed by occlusion, resulting in the perception of a fragmentary element in its complete form.^26,27,31^ This process seems to be one of the fundamental abilities that animal species, including human beings, have acquired through evolution. To accomplish perceptual completion of a partly occluded object, an organism has to either recognize or reconstruct it from its partial features.^28^ Which kind of recognition process is used by the organism depends on the context, genetics and adaptive characteristic of the given species.^29^ Human beings, for instance, attend to the global shape of the item rather than to its featural aspects to represent partly occluded objects in the visual system as complete forms.^30,31^ Conversely, research conducted on non-human animals (e.g., Japanese macaques, mice, and chicks) has demonstrated that these animals attend mainly to the featural aspects of the target.^32,33,47,50^ Further studies are needed to ascertain the extent to which this process is prevalent in living organisms and the way it has evolved. A crucial question, therefore, pertains to the potential extension of the perceptual completion process to plants. And, if such an extension exists, it is necessary to assess the sophistication of this process in relation to its observed in different animal species. Here, we aim to test whether plants are capable of such completion by splitting a support into two parts positioned at opposite locations. One part was entirely underground and therefore perceivable only by the roots. The other part was lifted from the ground, making it accessible only to the aerial part. The pea plants’ ascent and clasping behavior was video recorded to allow for 3D kinematical analysis. This methodology will allow to investigate and quantify if plants are able to integrate information derived by the presentation of a split support whose parts are located at opposite locations and to modify their behavior accordingly.

We hypothesize that if pea plants are able to integrate information derived from the in-ground and aboveground parts of the support, then they will orient themselves toward and clasp the support. The resulting kinematical pattern would reflect what has been previously observed when pea plants face an undivided support.^11,12,34^ Alternatively, if such binding does not occur, plants will likely exhibit a disoriented explorative behavior that does not lead to clasping the support. The kinematic pattern would align with what has already been reported when root access to the support is prevented.^25^

## Materials and methods

### Plant material

Fifty-two snow peas *(Pisum sativum* var. saccharatum cv Carouby de Maussane) were chosen as model plants (Table 1). *P. sativum* seeds were selected, potted and kept at the conditions outlined below (see *Germination and growth conditions* section).

### Types of support

**Figure 1:**
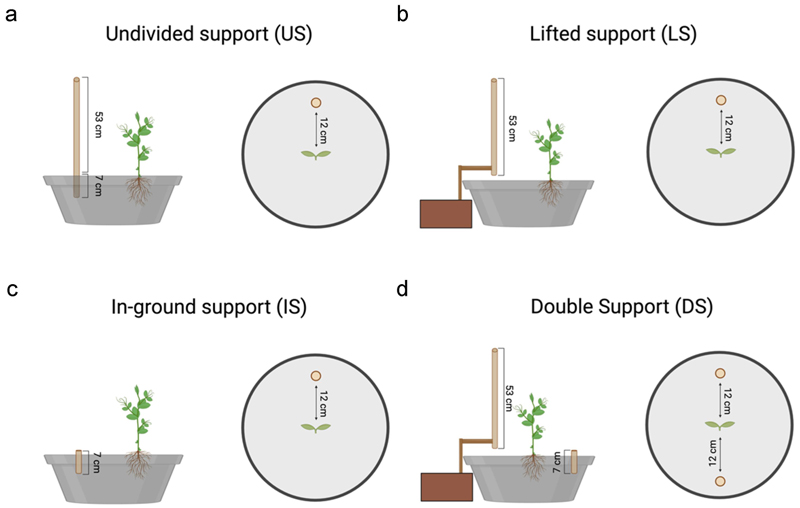
Graphical representation of the types of support used for each experimental condition.

Four types of support were considered:

(i) Undivided support: the support was a 60-cm tall wooden pole with a diameter of 1.2 cm, inserted 7 cm below the sand soil surface (Figure 1(a)).
(ii) Lifted support: the support was a 53-cm tall wooden pole with a diameter of 1.2 cm, lifted to the ground by means of an ad hoc apparatus (i.e., an external wooden extension; Figure 1(b)).
(iii) Grounded support: the support was a 7-cm tall wooden pole with a diameter of 1.2 cm, buried under the sand soil surface (Figure 1(c)). As a spatial reference, the support’s upper surface was visible to both cameras.

For all conditions, the support was positioned 12 cm away from the plant, and its location was counterbalanced in each experimental condition.

### Experimental conditions

Plants underwent four experimental conditions:

(A) *Undivided support condition* (*US*): pea plants were tested in the presence of a support whose below- and aboveground components were available to the root system and the aerial part, respectively (Figure 1(a)).
(B) *Lifted support condition* (*LS*): pea plants were tested in the presence of a lifted support that was available only via the aerial part of the plant (Figure 1(b)).
(C) *In-ground support condition* (*IS*): pea plants were tested in the presence of a grounded support available to the plant only via the root system (Figure 1(c)).
(D) *Double support condition* (*DS*): pea plants were tested in the presence of a lifted support available only via the aerial part of the plant and a grounded support available only via the root system. Supports were placed on opposite sides of the pot (Figure 1(d)).

### Germination and growth conditions

**Figure 2:**
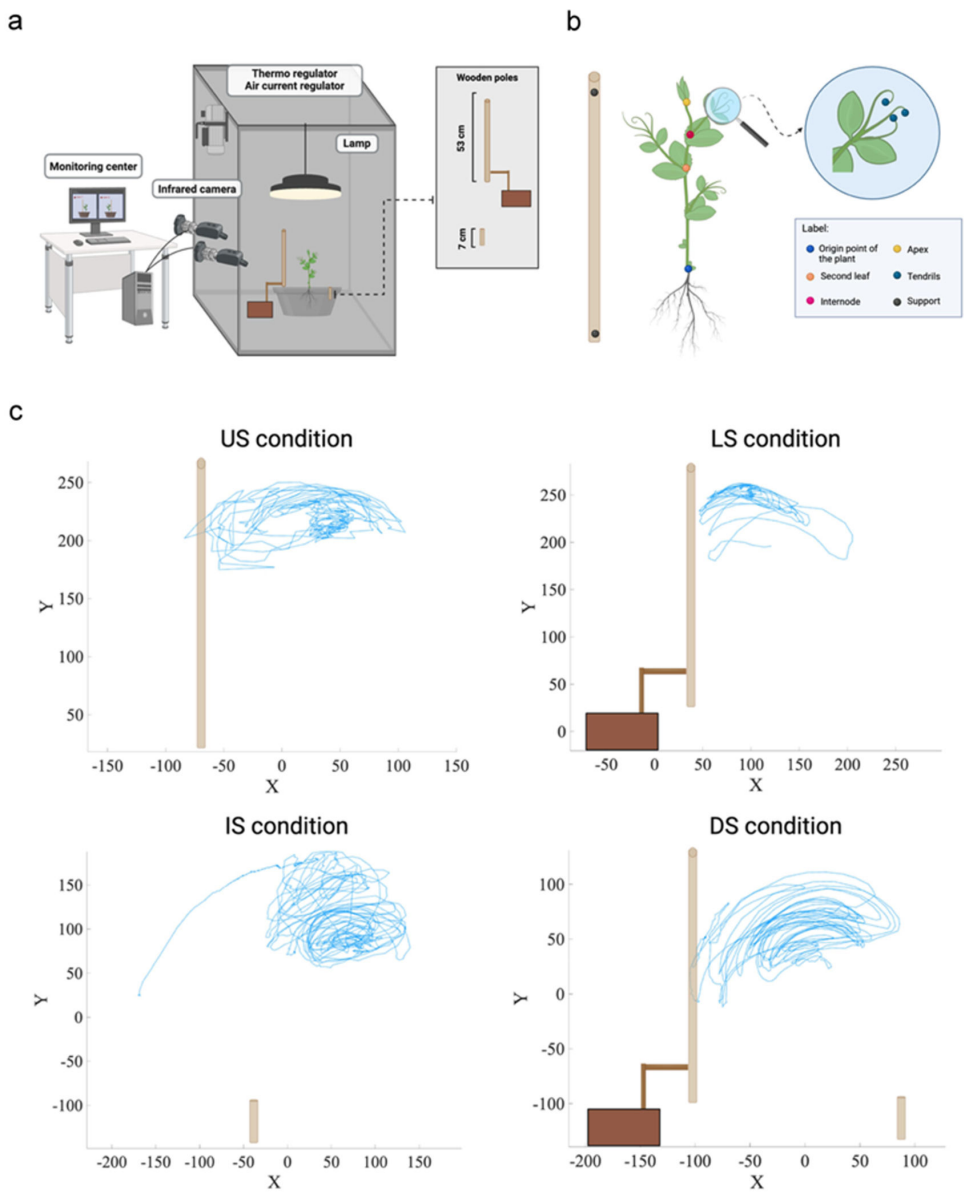
A. Graphical illustration of the experimental setup. b) Graphical representation of the anatomical landmarks of interest (the apex and the tips of the tendrils). Five reference points were also considered: the origin of the plant, the second leaf, the internode and the support’s lowest and the highest points. c) Representative trajectories for the tendril’s movement of the grasping leaf for the *US, LS, IS* and *DS conditions*. The x and y axes refer to the sagittal and vertical axis in mm, respectively. Please note that in the US and DS conditions, the tendrils clasp the support. This does not occur in the LS condition. In the is condition, the plants orient their movement toward the location of the support as presented in its entirety.

Cylindrical pots (diameter 30 cm, height 20 cm) were filled with silica sand (type 16SS, size 0.8/1.2 mm, density 1.4 kg l-1). The pots were watered and fertilized using a half-strength solution culture (Murashige and Skoog Basal Salt Micronutrient Solution; 10×, liquid, plant cell culture tested; SIGMA Life Science, Milan, Italy) three times a week from the plant transplanting into the pot until the end of the experimental session. Seeds were soaked in tap water for 24 hours and then placed in absorbent paper for 5 days to germinate in darkness at 26°C. Once the seeds germinated, healthy seedlings of the same root and shoot length were chosen and transplanted at the center of the pot 10 cm from each support. A growth chamber with controlled environmental conditions was used for each pot (Cultibox SG combi 80 × 80x160 cm; Figure 2(a)). The chamber air temperature was set at 26°C; the extractor fan was equipped with a thermo-regulator (TT125; 125 mm-diameter; max 280 MC/H vents), and there was an input-ventilation fan (Blauberg Tubo 100 - 102 m3/h). The two-fan combination allowed for a steady air flow rate in the growth chamber with a mean air residence time of 60 seconds. The fan was placed so that the air flow did not affect the plant’s movements. Plants were grown with a day/night cycle of 11h15’/12h45’under a cool-white LED lamp (V-TAC innovative LED lighting, VT-911-100W, Des Moines, IA, USA or 100 W Samsung UFO 145 lm/W – LIFUD) that was positioned 50 cm above each seedling. Photosynthetic Photon Flux Density at 50 cm under the lamp in correspondence with the seedling was 350 umolph/(m2s) (quantum sensor LI-190 R, Lincoln, Nebraska USA). Reflective Mylar^®^ film placed on the chamber walls allowed for better uniformity in light distribution. Each plant was tested individually in a single growing chamber. Treatments were replicated seven times by randomly assigning treatments to the eight growing chambers.

### Video recording and data analysis

For each growth chamber, a pair of RGB-infrared cameras (i.e., IP 2.1 Mpx outdoor varifocal IR 1080P) were placed 110 cm above the ground, 45 cm apart to record stereo images of the plant. The cameras were connected via Ethernet cables to a 10-port wireless router (i.e., D-link Dsr-250n) connected via Wi-Fi to a PC, and the frame acquisition and saving process were controlled using CamRecorder software (Ab.Acus s.r.l., Milan, Italy; Figure 2(a)). To maximize the contrast between the anatomical landmarks of the *P. sativum* plants (e.g., the tendrils) and the background, black felt velvet was fixed on some parts of the boxes’ walls and the wooden supports were darkened with charcoal. Each camera’s intrinsic and extrinsic lens distortion parameters were estimated using a Matlab Camera Calibrator App. Depth extraction from the single images was carried out by taking 20 pictures of a chessboard (18-mm squares, 10 columns, 7 rows) from multiple angles and distances in natural non-direct light conditions. For stereo calibration, the same chessboard used for the single-camera calibration process was placed in the middle of the growth chamber. The two cameras then took the photos to extract the stereo calibration parameters. In accordance with the experimental protocol, the cameras synchronously acquired a frame every 3 minutes (frequency 0.0056 hz). Ad hoc software (Ab.Acus s.r.l., Milan, Italy;^35^ developed in Matlab was used to position the markers and track their position frame-by-frame on the images the two cameras acquired to reconstruct each marker’s 3D trajectory. For each experimental condition, the leaf that coiled around the support or fell was considered in the analysis. The initial movement was defined as the frame in which the tendrils started to develop and were clearly visible from the apex. The end of the movement was defined as the frame in which tendril (s) started to wrap around the support or the frame before the plant fell. The markers on the plants’ anatomical landmarks of interest – namely the apex, the junction of the tendrils, and the tips of the tendrils – were inserted post hoc (Figure 2(b)). Markers were also positioned on the support (i.e., on the lowest and the highest points of the support), the origin of the plant, the second leaf and the internode as reference points (Figure 2(b)). The tracking procedures^35^ were first performed automatically throughout the movement sequence using the Kanade-Lucas-Tomasi algorithm on the frames each camera acquired, after distortion removal. The tracking was manually verified by the experimenter, who checked the marker’s position frame by frame. Each tracked marker’s 3D trajectory was computed by triangulating the 2D trajectories obtained from the two cameras (Figure 2(c)).^35^

### Kinematical dependent variables

The dependent variables specifically tailored to test our topic based on previous studies^11,12,25,34–39^ were:

(i) Movement time (min): the interval between the beginning and end of the tendrils’ movement (i.e., when the tendrils encountered the support or fell).
(ii) Average velocity (mm/min): the tendrils’ average velocity during circumnutation.
(iii) The peak of maximum velocity (%): the percentage of time at which the plant organ (i.e., apex or tendrils) reached the maximum peak velocity.
(iv) The duration of the circumnutation (min): the time a plant required to complete a single circumnutation.
(v) The total number of circumnutations the plant performed during the whole movement.
(vi) The average length of the circumnutation (mm): the mean value of the maximum distance between two points on the circumnutation’s trajectory.

### Statistical analysis

Data analyses were computed in the R environment.^40^ Data from the kinematical dependent variables were analyzed by means of the lmer function^41^ to perform linear mixed effect models with conditions as a between factor and plant’s ID as a random factor. The number of observations considered for each model was 115. Post hoc analyses were conducted using the pairwise contrast test of the emmeans R package^42^ in the presence of a statistically significant main effect. The significance level was set at *p* < 0.05.

## Results

### Qualitative results

**Figure 3:**
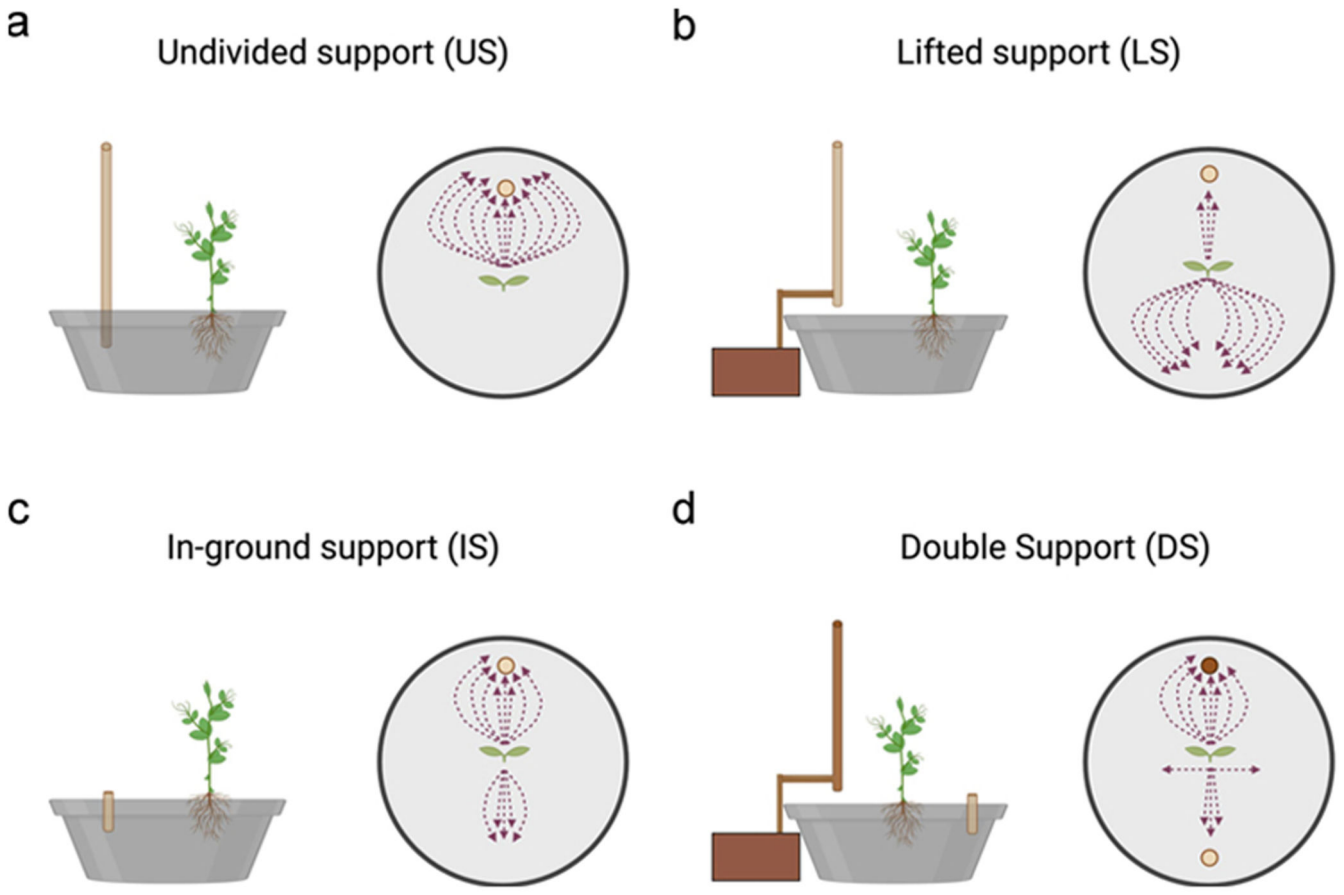
Graphical representation of the movement pattern of plants for the *US, LS, IS and DS* conditions. a) in the US condition all plants (13 out of 13) directed their approach and clasping movement toward the undivided support (i.e., the light brown circle). b) in the LS condition the majority of the plants (10 out of 13) were not able to localize the support, and they fell to the ground. c) in the is condition most of the plants directed their searching movement toward the side to which the buried support was located (8 out of 13). d) in the DS condition, most of the plants directed their movement toward the pot’s side in which the lifted support was located (i.e., the dark brown circle; 8 out of 13). Only a small part of the sample directed their movement toward the grounded support (i.e., the light brown circle; 3 out of 13) or they fell on an area not including either sector of the support. Please note that the trajectories of the plant’s movement are represented by means of the dashed lines.

The apex and the tendrils showed a growing movement pattern characterized by circumnutation (Figure 2(c)). In the *US condition*, once the plant perceived the support, it started to move toward it and the tendrils began to assume a choreography suited to grasp the support (13 out of 13; Video S1; Figure 3(a)).

When the support was lifted to the ground and therefore unavailable to the root system (*LS condition*), plants were not able to localize it except in a few cases (3 out of 13). In this condition, plants continued to move toward the light, and when they failed to find the potential support, they fell to the ground (10 out of 13; Video S2; Figure 3(b)). When the support was grounded and available only to the root system (*IS condition*), most of the plants directed their searching movement toward the side of the pot in which the grounded support was located (8 out of 13; Video S3; Figure 3(c)). The other plants fell in an area not corresponding to where the grounded support was located (5 out of 13; Figure 3(c)). In the presence of the split support (*DS condition*), most of the plants directed their searching movement toward the pot’s side in which the lifted sector was located (8 out of 13; Video S4; Figure 3(d)). Only a small part of the sample directed the aerial section toward the grounded element, which was available only to the root system (3 out of 13; Figure 3(d)). In some cases, the plants fell on an area not including either sector of the support (2 out of 13; Figure 3(d)).

**Figure 4:**
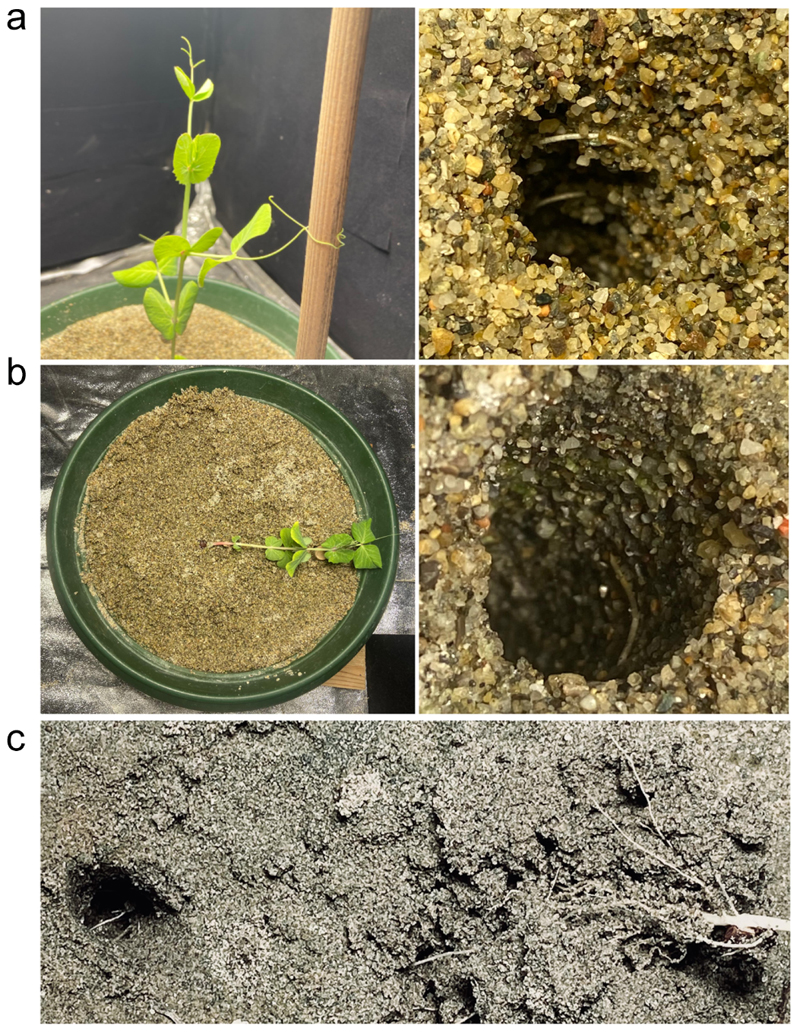
Photographs of the roots clinging to the belowground part of the support in the *US condition* (a) and the in-ground support for the *IS condition* (b). Panel C shows that in the *DS* condition, the roots twist around the in-ground part of the split support.

Considering the roots’ final location once the plant either grasped the support or fell, in the *US condition* (Figure 4(a)), the roots were twisted around the inground part of the support. In this condition, the plant performed a successful clasping behavior. In the *IS condition* (Figure 4(b)), the same occurred, but because above-ground there was no support, the plants fell. Noticeably, the majority of plants fell at the location of the in-ground support. In the *DS condition* (Figure 4(c)), it is evident that the roots traveled toward the in-ground part of the support. In the *LS condition*, it was difficult to ascertain the root’s final position because the belowground part of the support was not present; therefore, the plants did not move toward and coil around it. They were sparse all around the sand soil.

### Kinematical results

At the kinematical level, a significant main effect of the type of support for the average velocity, the peak of maximum tendril velocity, movement time duration, duration of the circumnutation, length of the circumnutation and the number of circumnutations was found (Table 2). Comparisons in each condition are set out in the following subsections.

### US vs LS condition

**Figure 5:**
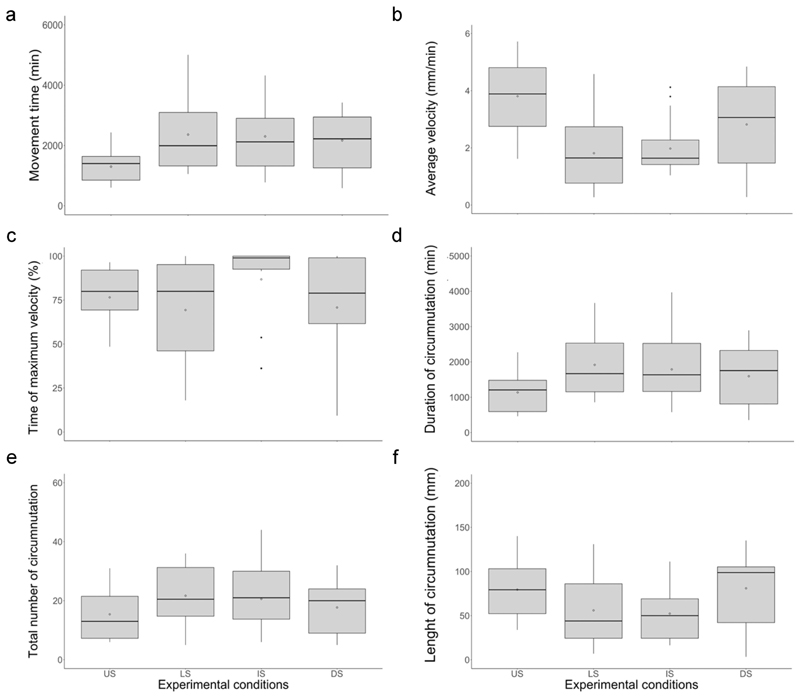
Box plot representing the values for movement time (a), the average velocity, (b) the time of maximum velocity (c), the duration of circumnutation (d), the number of circumnutation (e) and the length of circumnutation (f) of the tendrils in the *US, LS, IS* and *DS conditions*. Boxplots show the 25th and 75th percentiles; the horizontal bar in the box plot represents the median; the diamond represents the mean; the black dots indicate outliers.

This comparison allows for the establishment of the implications of the roots not perceiving the support. In the comparison of the kinematics of the tendrils of pea plants between the *US* and *LS conditions* (Figure 1(a,b)), post hoc comparisons showed that movement time (Figure 5(a) and Table 3) and the duration of circumnutation (Figure 5(d) and Table 3) were shorter, the average tendril velocity was higher (Figure 5(b) and Table 3) and the total number of circumnutations was lower (Figure 5(e) and Table 3) in the *US* than in the *LS condition*. No significant differences between these conditions for the peak of maximum tendril velocity (Figure 5(c) and Table 3) or length of circumnutation (Figure 5(f) and Table 3) were observed.

### US vs IS condition

This comparison allows for the establishment of the consequences of the plant not perceiving the upper part of the support. Post hoc comparison of the kinematics of the tendrils of pea plants between the *US and IS conditions* (Figure 1(a,c)) showed that movement time (Figure 5(a) and Table 3) and the duration of circumnutation (Figure 5(d) and Table 3) were shorter, the average velocity (Figure 5(b) and Table 3) was higher and the length of the circumnutation (Figure 5(f) and Table 3) was shorter in the *US* than in the *IS condition*. No significant differences between these conditions for the peak of maximum tendril velocity (Figure 5(c) and Table 3) and the total number of circumnutation (Figure 5(e) and Table 3) were observed.

### LS vs IS conditions

This comparison allows us to determine the consequences of the plant perceiving only a single part of the support, whether the lower or upper part of it. Post hoc comparison of the kinematics of the tendrils of pea plants in the *LS* and *IS conditions* (Figure 1(b,c)) showed that the peak maximum velocity (Figure 5(c) and Table 3) occurred later in the *IS* than in the *LS condition*. No significant differences between these conditions for the movement time (Figure 5(a) and Table 3), the average tendril’s velocity (Figure 5(b) and Table 3), the duration of circumnutation (Figure 4(d) and Table 3), the total number of circumnutations (Figure 5(e) and Table 3) or the length of circumnutation (Figure 5(f) and Table 3) were observed.

### US vs DS condition

This comparison allows us to determine the consequences of splitting the support so that its lower and the upper parts were located at the opposite sides of the pot. Post hoc comparison of the kinematics of the tendrils between the *US* and the *DS condition* (Figure 1(a,d)) showed that movement time was longer (Figure 5(a) and Table 3) and the average tendril velocity was lower (Figure 5(b) and Table 3) in the divided- than the undivided-support condition. No significant differences between these conditions for the peak of maximum tendril velocity (Figure 5(c) and Table 3), the duration of circumnutation (Figure 5(d) and Table 3), the total number of circumnutations (Figure 5(e) and Table 3) or the length of circumnutation (Figure 5(f) and Table 3) were observed.

## Discussion

The present study was conducted to ascertain whether pea plants are able to face situations in which a potential support cannot be perceived in its entirety and to perform a functional approach-to-clasp movement toward it. To this end, we split a support in two parts at various locations. One part is perceivable only by the roots, and the other is perceivable only by the plants’ aerial section. Our findings showed that pea plants were able to use the information at either end to successfully complete the ascent and clasping behaviors.

At first, our findings’ reliability is evidenced by the fact that we were able to confirm previous observations suggesting that lifting the support from the ground, thus preventing the roots’ access to the support, caused a failure in detecting and clasping the support.^25^ The plant appears to be in a state of spatial uncertainty, slowing down the tendrils and prolonging the length of circumnutation when compared to the condition in which the support was presented in its entirety. The same scenario occurs when the in-ground part of the divided support presented in isolation was compared with the entire support (*US condition*). These findings may indicate that when the roots or the aerial part of the plant are the only “actors” involved in the perceptual processing of the support, then plants adopt a compensatory strategy characterized by a more cautious circumnutation pattern. However, this approach proves unsuccessful in achieving effective support clasping. Collectively, these results indicate that perceiving only a single part of the support, whether the lower or upper part, does not allow for the organization of a functional ascent and clasping movements in pea plants, highlighting the roots’ crucial role in sensing the support’s location, as shown in Figure 3.

Turning to the main issue at the core of the present study, the fact that in the *DS condition* the plants are able to orient themselves and clasp the support seems to suggest that they are able to resolve the mismatch caused by the two parts of the support being positioned in different locations. The comparison between the DS and the US conditions revealed that plants exhibited reduced movement velocity and a prolonged movement time when the two parts of the support were positioned at opposing locations. A more cautions kinematical pattern may allow plants to process adequately the information coming from each target for establishing an accurate unified percept of the support.^37,38^ That is, the time required by plants to perform an approaching and clasping movement is proportional to the amount of information necessary for controlling the behavior as a function of task difficulty.^37,38,43,44^ Overall findings suggest that plants possess the capacity to process the location of the support and exhibit a form of perception that underpins anticipatory behavior.^12^ The key question is how plants integrate information coming from different spatial locations. With a great degree of caution, we are tempted to interpret present findings according to a phenomenon termed “perceptual completion”.^27,28,31^ Perceptual completion is a process of bridging the gaps created by partial occlusion of an object, allowing an organism to perceive it as a complete unit.^26,27,31^ This perceptual effect occurs when two parts of an interrupted object are matched based on spatiotemporal and featural information continuity.^45,46^ This effect is also observed across various animal species,^33,47^ such as chimpanzees,^48^ rhesus monkeys,^29^ baboons,^32^ mice^49^ and chicks.^50,51^ A comparable perceptual phenomenon may also occur in plants under our *DS condition*. The simultaneous presentation of two complementary items with similar shapes and materials may have resulted in the perception of the two parts as a single entity, influencing the plants’ searching. To solve the correspondence problem and clarify the ambiguous input resulting from the mismatch in the two supports’ locations, plants may rely on information the root system and the aerial part provided. This information is interpreted and integrated to achieve a unified perception of the targe element (i.e., support). How this can be achieved remains is a challenge, but we are tempted to think that each of the plant’s modules could, in principle, detect and react to their surroundings by interpreting subtle variations in electromagnetic radiation, far-red light reflection, nutrient availability and various chemicals other organisms produce.^52,53^ If the properties of the supports (e.g., size, texture, material) are similar, then on the basis of such similarities plants might, somewhat, unify them. If this is the case, the strategy would be analogous to that observed in non-human animals for achieving perceptual completion of a partially occluded object.^33,47,54^ Overall we advance that the process of support integration might take place in two phases. First, the root system might acquire preliminary information about the support’s position through root exudates^15^ and/or mechanical stimulation^1,55,56^ (Figure 3). Then, this information could be transmitted to the aerial part of the plant, where it is integrated with the proprioceptive feedback accumulated during each single leaf’s circumnutation.^11,57^ At the physiological level, interactions between the roots and aerial parts of the plant are mediated by a variety of signaling molecules, including plant hormones such as auxins, cytokinins, strigolactones, gibberellins, and abscisic acid (ABA), which play a crucial role in regulating growth and development.^58,59^ These hormonal molecules are transported from the roots through the phloem and xylem, enabling effective communication between the roots and shoots.^60^ Root-derived cytokinins and strigolactones play pivotal roles in coordinating shoot development based on nutrient status^61–63^ whereas auxin and gibberellins facilitate shoot-to-root communication and long-distance coordination in the plant.^59^ Additionally, ABA and jasmonates act as critical mediators in root-to-shoot signaling under drought and salt stress conditions.^64^ For instance, recent findings suggest that lipid-binding and transfer proteins may enhance the solubility of hydrophobic hormones such as ABA and jasmonates, supporting their movement through the plant’s vascular tissues.^65^ The intricate balance between these signaling pathways enables plants to adapt their growth in response to resource availability, thus optimizing development under variable environmental conditions.^66,67^ This complex signaling network may therefore serve as a mechanism for perceptual completion in plants. By integrating fragmented spatial information from root and shoot systems, plants may be able to solve the mismatch in the position of the two parts of the support, guide their search behavior and reorganize the kinematics of the approach to grasp-movement properly.^14,19–24^ Another possible explanation is the well documented involvement of mechanosensitive calcium channels (MS channels) in root responses to physical stimuli. These channels, particularly in root cap cells, play a crucial role in detecting obstacles in the soil and mediating rapid signaling events.^68–70^ When roots encounter a physical barrier, a rapid and transient increase in cytosolic calcium ([Ca^2 +^]cyt) occurs,^71^ and these calcium signals are often specific to the type and intensity of the mechanical stimulus.^72^ These [Ca^2 +^]cyt fluctuations act as key second messengers, triggering downstream cascades involving calcium-binding proteins that regulate gene expression and physiological responses.^73^ In the context of our study, it is plausible that the buried segment of the support activates MS channels in the root, leading to a calcium influx and initiating a systemic signal toward the shoot. This signal could accelerate the plant’s response without necessarily involving the integration of sensory information into a coherent perception of the object. Mechanical signals are known to be transmitted over long distances via electrical signals, hydraulic changes, or calcium waves propagating through plant tissues.^74^ The spatiotemporal patterns of calcium signals – referred to as “calcium signatures” - are crucial for encoding specific responses.^73^ Moreover, the cytoskeleton and reactive oxygen species (ROS) production are often associated with these calcium-mediated signaling processes, further modulating root growth and development.^75^ In this context, it is conceivable that the buried segment of the support could activate MS channels in the root, triggering a calcium signal that propagates toward the shoot and accelerates the plant’s response. This signaling pathway would not require the integration of sensory information into a unified representation of the support but could instead rely on localized stimuli perceived at different contact points. Calcium waves and electrical signals are known to mediate long-distance communication between roots and shoots, contributing to systemic responses.^74^ While this mechanosensitive signaling mechanism does not exclude the perceptual completion hypothesis, it highlights a complementary process that could coexist and contribute to the plant’s rapid adaptation. Future experiments focusing on calcium imaging and the use of mutants with impaired MS channel function could help unravel the precise role of these channels and their potential interaction with other signaling pathways.

It should be noted, however, that the present study is not without limitations. To start with, the observation of the root’s location at the conclusion of the experiment remained at a qualitative level. Further studies should include the measurement of the morphological characteristics of the plant’s roots (i.e., the length of primary roots, the fresh and dry weight of root mass) and aerial component (e.g., stem height and tendril length). This would allow for the investigation of whether the perception (or not) of the belowground portion of the support may also affect the plant’s morphology. Additionally, it would be necessary to ascertain which of the plant’s components establishes the first contact with the support. In other words, it remains unclear whether the roots initially touch the support enabling the aerial portion to grasp it or this process occurs in a reverse order or simultaneously. By assessing this aspect, it would be possible to define the hierarchy of the organization of the approach-to-clasp movement in pea plants and gain new insight into the growth patterns among plants. Moreover, our study is limited to a specific variety of pea plant (*Pisum sativum* var. saccharatum cv. Carouby de Maussane). Therefore, our findings may not be extended to the entire category of pea plants or to climbing plants in general. Therefore, it would be necessary to conduct other studies on various pea genotypes (e.g., *Pisum sativum* var. sativum, *Pisum sativum* var. macrocarpon, etc.) and/or other climber species, such as the bean plant (*Phaseolus vulgaris* L.). By testing our hypotheses in multiple samples, we may ascertain whether this behavior is a general phenomenon among climbing plants or is an adaptive behavior specific to a certain plant species. Finally, the phenomena investigated here should be evaluated in natural crowded settings, where potential supports are often partially occluded for climbers. Studies in a natural setting are necessary to verify these findings’ relevance in an ecological context. The fact that a plant responds in a particular way in an unnatural environment does not necessarily mean it will do so in the natural one. Integrating these results with ecological observations could help identify specific factors related to support sensing in climbing plants.

## Conclusions

The present findings confirmed and extended previous evidence of the existence of crosstalk between the above- and the belowground parts of pea plants subtending the support detection process. They suggest that the root system plays a pivotal role in sensing the presence of a support and that the information perceived affects the execution of our plants’ ascent and clasp behavior. Researchers should investigate this endeavor’s physiological components to gain further insights into the functional equilibrium and interactivity between plants’ modules and to expand our understanding of plants’ various behavioral strategies in response to environmental cues.

## Supplementary Material

Supplementary Material

## Figures and Tables

**Table 1:** Sample description.

Undivided support (US)	
N°	13
Distance from the support	12 cm
Age	15 d (±0.61; range 10–21)
***Lifted support (LS)***	
N°	13
Distance from the support	12 cm
Age	28 d (±4; range 20–39)
***In-ground support (IS)***	
N°	13
Distance from the supports	12 cm
Age	13.5 d (±5; range 6–18)
***Double support (DS)***	
N°	13
Distance from the supports	12 cm
Age	8 d (±4.39; range 7–25)

The age, which is expressed in days, refers to the period from the transplanting of the seedling into the pot until the clasping of the support (e.g., *US* and/or *DS condition*) or the falling of the plant (e.g., *LS* and/or *IS condition*). The age refers to the median, and median absolute deviation is noted in parentheses.

**Table 2:** Results from the lmer fitted models (type III Wald chi-square tests) investigating the differences across experimental conditions (*US, LS, IS* and *DS*) for the kinematical variables considered.

	χ^2^	df	Pr(>χ^2^)	R^2^
**Average velocity (mm/min) ~**				
(Intercept)	125.129	1	<0.001***	
Condition	56.163	3	<0.001***	
Marginal R^2^				0.330
Conditional R^2^				0.349
**Time of peak max. velocity (%) ~**				
(Intercept)	140.430	1	<0.001***	
Condition	16.193	3	0.001**	
Marginal R^2^				0.107
Conditional R^2^				0.333
**Movement time (min) ~**				
(Intercept)	82.664	1	<0.001***	
Condition	24.118	3	<0.001***	
Marginal R^2^				0.152
Conditional R^2^				0.395
**Duration of circumnutation (min)** **~**				
(Intercept)	76.068	1	<0.001***	
Condition	17.964	3	<0.001***	
Marginal R^2^				0.127
Conditional R^2^				0.284
**Length of circumnutation (mm) ~**				
(Intercept)	103.689	1	<0.001***	
Condition	13.781	3	0.003**	
Marginal R^2^				0.104
Conditional R^2^				0.203
**Total number of circumnutation ~**				
(Intercept)	67.955	1	<0.001***	
Condition	8.573	3	0.035*	
Marginal R^2^				0.062
Conditional R^2^				0.248

χ^2^ = Chi-squared test, df = Degrees of Freedom, R^2^ = Coefficient of determination, * = *p* < 0.05, ** = *p* < 0.01, *** = *p* < 0.001. mm = millimeter, min = minutes, % = percentage.

**Table 3:** Post hoc analysis (emmeans contrast) of the significant effects detected in the previous four models.

	estimate	SE	df	t ratio	*p*-value
**Average velocity (mm/min) ~**					
IS – US	−1.78	0.30	111	−6.004	<0.001***
IS – LS	0.17	0.28	110	0.599	0.932
US – LS	1.95	0.29	111	6.665	<0.001***
DS – IS	0.82	0.33	110	2.503	0.065
DS – US	−0.96	0.33	111	−2.916	0.022*
DS – LS	0.99	3.19	104	3.117	0.012*
**Time of max velocity (%) ~**					
IS – US	10.91	6.06	109.2	1.800	0.279
IS – LS	21.74	5.72	103.1	3.802	0.001**
US – LS	10.83	5.94	108.0	1.824	0.268
DS – IS	−19.00	6.60	105.9	−2.880	0.024*
DS – US	−8.09	6.65	107.7	−1.217	0.618
DS – LS	2.74	6.23	98.9	0.440	0.971
**Movement time (min)~**					
IS – US	949.3	243	108.5	3.903	<0.001***
IS – LS	−110.3	229	102.3	−0.482	0.963
US – LS	−1059.6	238	107.3	−4.449	<0.001***
DS – IS	−41.1	264	104.9	−0.156	0.999
DS – US	908.2	267	107.0	3.406	0.005*
DS – LS	−151.4	249	98.5	−0.609	0.929
**Duration of circumnutation (min) ~**					
IS – US	621	201	110.4	3.085	0.013*
IS – LS	−179	191	105.0	−0.935	0.786
US – LS	−800	191	109.2	−4.050	<0.001***
DS – IS	−143	220	108.1	−0.650	0.915
DS – US	478	221	109.0	2.159	0.141
DS – LS	−800	209	99.8	−1.542	0.417
**Length of circumnutation (mm)~**					
IS – US	−28.093	8.77	111	−2.634	0.047*
IS – LS	−0.829	8.40	107	−0.099	1.000
US – LS	22.264	8.62	110	−2.583	0.053
DS – IS	24.796	9.64	110	2.530	0.055
DS – US	1.703	9.66	110	−0.529	0.998
DS – LS	23.967	9.22	101	2.621	0.051
**Total number of circumnutation ~**					
IS – US	4.40	2.37	110.1	1.857	0.253
IS – LS	−2.12	2.24	104.5	−0.945	0.781
US – LS	−6.52	2.32	108.9	−2.808	0.030*
DS – IS	−1.77	2.59	107.5	−0.686	0.902
DS – US	2.62	2.60	108.7	1.008	0.745
DS – LS	−6.52	2.45	99.5	−1.590	0.389

IS = In-ground support condition, LS = Lifted support condition, US = Undivided support condition, DS = Double support condition, SE = Standard Error, df = Degrees of Freedom.

* = p < 0.05,

** = p < 0.01,

*** = p < 0.001.

mm = millimeter, min = minutes, % = percentage.

## Data Availability

All data presented in the study are openly available in Zenodo at: https://doi.org/10.5281/zenodo.14164665.
